# Abrogation of the RNase activity of E^rns^ in a low virulence classical swine fever virus enhances the humoral immune response and reduces virulence, transmissibility, and persistence in pigs

**DOI:** 10.1080/21505594.2021.1959715

**Published:** 2021-08-02

**Authors:** Miaomiao Wang, José Alejandro Bohórquez, Yoandry Hinojosa, Sara Muñoz-González, Markus Gerber, Liani Coronado, Carmen Laura Perera, Matthias Liniger, Nicolas Ruggli, Llilianne Ganges

**Affiliations:** a OIE Reference Laboratory for Classical Swine Fever, IRTA-CReSA, Barcelona, Spain; b Division of Virology, Institute of Virology and Immunology IVI, Mittelhäusern, Switzerland; c Department of Infectious Diseases and Pathobiology (DIP), Vetsuisse Faculty, University of Bern, Bern, Switzerland; d Graduate School for Cellular and Biomedical Sciences (GCB), University of Bern, Bern, Switzerland; e Centro Nacional De Sanidad Agropecuaria (CENSA), Mayabeque, Cuba

**Keywords:** Classical swine fever virus (CSFV), E^rns^ RNase activity, viral replication, type I IFN, viral attenuation, viral persistence, viral transmission, humoral response, *Pestivirus*

## Abstract

The prevalence of low virulence classical swine fever virus (CSFV) strains makes viral eradication difficult in endemic countries. However, the determinants for natural CSFV attenuation and persistence in the field remain unidentified. The aim of the present study was to assess the role of the RNase activity of CSFV E^rns^ in pathogenesis, immune response, persistent infection, and viral transmission in pigs. To this end, a functional cDNA clone pPdR-H_30_K-36U with an E^rns^ lacking RNase activity was constructed based on the low virulence CSFV field isolate Pinar de Rio (PdR). Eighteen 5-day-old piglets were infected with vPdR-H_30_K-36U. Nine piglets were introduced as contacts. The vPdR-H_30_K-36U virus was attenuated in piglets compared to the parental vPdR-36U. Only RNA traces were detected in sera and body secretions and no virus was isolated from tonsils, showing that RNase inactivation may reduce CSFV persistence and transmissibility. The vPdR-H_30_K-36U mutant strongly activated the interferon-α (IFN-α) production in plasmacytoid dendritic cells, while *in vivo*, the IFN-α response was variable, from moderate to undetectable depending on the animal. This suggests a role of the CSFV E^rns^ RNase activity in the regulation of innate immune responses. Infection with vPdR-H_30_K-36U resulted in higher antibody levels against the E2 and E^rns^ glycoproteins and in enhanced neutralizing antibody responses when compared with vPdR-36U. These results pave the way toward a better understanding of viral attenuation mechanisms of CSFV in pigs. In addition, they provide novel insights relevant for the development of DIVA vaccines in combination with diagnostic assays for efficient CSF control.

## Introduction

Classical swine fever (CSF) is one of the oldest and most studied diseases in animal health and continues to be a threat in the porcine sector worldwide [1]. The causative agent, classical swine fever virus (CSFV), is an enveloped RNA virus that belongs to the *Pestivirus* genus within the *Flaviviridae* family. The CSFV genome is a single-stranded, positive-sense RNA of 12.3 kb carrying one long open reading frame (ORF). The ORF is flanked by a 5´- and a 3´-untranslated region (UTR) and encodes a polyprotein that is cleaved into four structural (C, E^rns^, E1, and E2) and eight nonstructural proteins (N^pro^, p7, NS2, NS3, NS4A, NS4B, NS5A, and NS5B) [2,3]. Among these proteins, E2 and E^rns^ are the main targets for neutralizing antibodies and induce protective immunity in infected animals [4,5].

The E^rns^ glycoprotein, exclusively present in pestiviruses, has a unique feature of intrinsic ribonuclease (RNase) activity among all viral envelope proteins [6]. This function is not essential for virus replication in tissue culture [7–9]. E^rns^ was reported to induce apoptosis in lymphocytes *in vitro*, and its RNase activity is required to prevent type I interferon (IFN) induction [10,11]. Studies in cell culture have shown that E^rns^ can degrade viral single- and double-stranded RNA both in the extracellular space and in endocytic compartments [12–14]. Interestingly, E^rns^ inhibits Toll-like receptor (TLR)7-dependent IFN-α induction in plasmacytoid dendritic cells (pDC) after direct CSFV infection or when activated through contact with CSFV-infected cells [11]. Nevertheless, the *in vivo* implications of these mechanisms has not been demonstrated yet. In the case of bovine viral diarrhea virus (BVDV), another important member of the Pestivirus genus, this function may help to establish and maintain persistent infections in cattle [10,15]. Previous studies showed that abrogation of the E^rns^ RNase activity in virulent pestiviruses including CSFV may reduce replication and clinical signs *in vivo* [8,9]. However, the role of the E^rns^ RNase in CSFV pathogenesis, immune response, and its relationship with the viral ability to generate chronic and persistent infections, have been scarcely studied.

It was shown previously that the low virulence CSFV field isolate Pinar de Rio (PdR) that resulted from natural CSFV evolution under endemic conditions [16] could lead to chronic and persistently infected piglets [17,18]. In addition, the unique uninterrupted poly-uridine (poly-U) sequence found in the 3´UTR of this isolate was identified as a new virulence factor that could activate immunity and attenuate virulence in piglets [19]. This previous study pointed towards a possible role played by the long poly-U sequence in the activation of innate immunity, which may be controlled by the RNase activity of E^rns^ [11]. Therefore, the present study focused on investigating the role of the RNase activity of E^rns^ *in vivo*. To this end, we generated the RNase-negative E^rns^ mutant CSFV vPdR-H_30_K-36U using the functional cDNA backbone pPdR-36U described previously [19]. We assessed virus replication and type I IFN responses *in vitro* and *in vivo* and the role of the E^rns^ RNase activity for viral pathogenicity, persistence, and transmission in pigs.

## Materials and methods

### Cells and viruses

The PK-15 cell line (ATCC CCL-33), the porcine aortic endothelial cell line PEDSV.15 [20] (obtained from Jörg Seebach, University of Geneva, Switzerland) and the SK-6 cell line [21] (obtained from M. Pensaert, Faculty of Veterinary Medicine, Ghent, Belgium) were tested for absence of pestiviruses. The PK-15 cells were cultivated in minimum essential medium (MEM) containing 10% pestivirus-free fetal bovine serum (FBS) and the two other cell lines were grown in Dulbecco’s Modified Eagle Medium (DMEM) containing sodium pyruvate, non-essential amino acids, and 7% horse serum. The PEDSV.15 were supplemented with an additional 2% porcine serum. Porcine monocyte-derived macrophages (MDM) and CD172a^+^ enriched pDC were prepared as described previously [11,19,22].

The CSFV vPdR-36U was rescued from the cDNA clone pPdR-36U [19]. This clone corresponds to the isolate Pinar del Rio (PR-11/10-3) from the Cuban CSF epizootic in 2010 [16,23]. PR-11/10-3 is also referred to as CSF1058 according to the nomenclature of the European Union Reference Laboratory for Classical Swine Fever (EURL-CSF), Hannover, Germany. The CSFV Alfort/187 strain, also provided by the EURL-CSF was used in the virus neutralization assay. The cDNA-derived viruses were rescued as described below. All viruses were amplified by infecting cells with 0.1 tissue culture infectious dose (TCID_50_)/cell and were harvested after 72 hours. End-point dilution was used to determine the virus titers in SK-6, PEDSV.15, and porcine MDM cells using 96-well tissue culture plates and the peroxidase‐linked assay (PLA) [24] with the monoclonal antibody (mAb) HC/TC-26 [25] against E2. The virus titer was expressed in TCID_50_/ml [26].

### Construction of the infectious clone pPdR-H_30_K-36U

The construction of the functional cDNA clone pPdR-H_30_K-36U encoding RNase-inactive E^rns^ was based on the plasmid backbone pPdR-36U cDNA described previously [19]. The pPdR-36U plasmid was modified by substitution of the histidine codon (cac) at amino acid position 30 of E^rns^ (amino acid 297 of the ORF, GenBank accession number KX576461) with a lysine codon (aaa). To this end, standard PCR-based mutagenesis was applied, and the PCR fragment carrying the mutation was used to replace the *Cla*I-*Kas*I cassette in pPdR-36U-5ʹh described previously [19], resulting in pPdR-H_30_K-5ʹh. The plasmid pPdR-H_30_K-36U was then obtained by insertion of the *Spe*I to *PspX*I cassette of pPdR-36U (from nucleotide position 8274 to the end of the genome including the *Srf*I run-off site) in the corresponding sites of pPdR-H_30_K-5ʹh. The construct was verified by Sanger sequencing.

### Virus rescue from cDNA

The vPdR-H_30_K-36U virus was rescued from cDNA as described previously for vPdR-36U [19]. Briefly, the plasmid was linearized with *Srf*I and RNA was transcribed with the MEGAscript T7 kit (ThermoFisher Scientific). The RNA was treated with DNase I and purified on S-400HR columns (GE Healthcare Life Sciences). 1 µg RNA was then mixed with 8 × 10^6^ PEDSV.15 cells in 0.4 ml ice-cold phosphate-buffered saline (PBS). The mix was transferred to a 0.2 cm electroporation cuvette, and electroporation was performed using a Gene Pulser (Bio-Rad) by applying 2 pulses at 200 V and 500 µF. The cells were then cultured in 75 cm^2^ flasks at 37°C and 5% CO_2_ during 65 hours. The rescued virus was passaged once into PEDSV.15 cells. In order to control the functionality of the constructs, the infectivity of the RNA transcripts was determined with an infectious center assay as described previously [27]. The complete genome sequences of the rescued viruses were verified by nucleotide sequencing to exclude any accidental mutation. Finally, viral titers were determined in PEDSV.15 and porcine MDM [19].

### Virus replication in cell culture

For the analysis of virus replication kinetics in cell culture, PEDSV.15 (150,000/well), and porcine MDM (500,000/well) were infected, respectively, in quadruplicate and in triplicate with vPdR-H_30_K-36U or vPdR-36U at a multiplicity of infection (MOI) of 0.02 TCID_50_/cell (according to titers from the homologous cell type) in 24-well plates using serum-free medium. The inoculum was removed after 1 h at 37°C and the cells were washed once with a serum-free medium followed by incubation in a complete culture medium at 37°C. Virus was harvested at the indicated hours post infection (h.p.i.) after one freeze/thaw cycle. The virus titer was determined by endpoint dilution in SK-6 cells.

### RNase activity assay

The RNase activity assay was performed as described previously [11,28]. Briefly, SK-6 cells were infected in 6-well plates (10 cm^2^ wells) with vPdR-36U or vPdR-H_30_K-36U or were mock infected or left uninfected. After 24 hours of incubation in complete medium, the cells were washed 5 times with PBS and cultured for additional 20 hours in serum-free MEM. Cell extracts were obtained by hypotonic lysis with H_2_O (400 µl per well). A 50-mer RNA probe (Dy-781-O1-RNA) labeled with Dyomics 781 at the 5ʹend (prepared by Dr. Fabian Axthelm, Microsynth AG, Balgach, Switzerland) was mixed at 40 nM with the cell extracts to be tested for RNase activity. RNase A (3 × 10^−3^ U/ml in MEM) was included as digestion control, and 50 mM TrisHCl pH 7.4 served as negative control. The reactions were incubated for 1 h at 37°C. Subsequently, two volumes of 97% formamide (Sigma) were added to the treated probes and a 10% polyacrylamide and 35% urea gel in 133 mM TrisHCl, 45.5 mM boric acid, and 3.2 mM EDTA were used for separation. The Odyssey Infrared Imaging System (LI-COR) was employed for image acquisition.

### Stimulation of pDC by infected cells

Enriched porcine pDC were stimulated with infected cells for IFN-α production as described earlier [11]. Briefly, porcine MDM were infected at a MOI of 5 TCID_50_/cell in 24-well plates (200ʹ000 cells/well) and cultured for 24 hours. CpG D32 at 5 µg/ml was used as a positive control for pDC stimulation. The cells were then washed four times to remove the inoculum, and 10^6^ freshly isolated CD172a^+^-enriched pDC were added to each well. After another 22 hours, the supernatants were harvested and analyzed for IFN-α by enzyme-linked immunosorbent assay (ELISA). The infection of the MDM was verified and quantified by optical density at 450 nm (OD_450_) using a CSFV E2-specific immunoperoxidase assay with mAb HC/TC-26. Means and standard deviations were calculated from six parallel cultures.

### IFN-α detection by ELISA

IFN-α in cell supernatants and sera samples was quantified by ELISA as described previously, using the anti-pig IFN-α K9 and the F17 mAbs (provided by Dr. B. Charley, INRA, Jouy-en-Josas, France) [29,30]. Serial dilutions of recombinant IFN-α protein (PBL Biomedical Laboratories, Piscataway, New Jersey, USA) were employed as a standard. Optical densities obtained with the cytokine standard were used in a regression curve for the determination of the cytokine concentrations (units/ml) in samples.

### Experimental infection

The experimental infection was performed following the protocol applied for the parental RNase-positive vPdR-36U virus used as control in the present study and described previously [19]. Twenty-seven piglets at 5 days of age, born from pestivirus-free sows from the same farm as in the previous study, were allocated in a box of the animal biosafety level 3 (aBSL3) facilities at IRTA-CReSA (Barcelona, Spain). The piglets (n = 18, numbered 1–18) were inoculated intranasally with 2.5 × 10^4^ TCID_50_ of vPdR-H_30_K-36U (according to the titer in PEDSV.15 cells). At 24 hours after infection, nine piglets (numbered from 19 to 27) were introduced as contact animals. Serum as well as nasal and rectal swabs were collected from the inoculated group at 7 days post infection (dpi). Subsequently, these samples were collected from all animals weekly until the end of the trial (5 weeks after infection). In addition, tonsil samples were obtained after euthanasia.

As described previously, a veterinarian monitored the animals daily in a blinded manner [31]. For ethical reasons, the animals were euthanized when they developed moderate to high clinical signs, including fall of the hindquarters, inability to drink or feed, prostration, or moderate to severe nervous disorders. Euthanasia was carried out according to the European Directive 2010/63/EU, with 60–100 mg/kilogram of bodyweight of pentobarbital injected into the *vena cava cranialis*. The Ethics Committee of the Generalitat of Catalonia approved the experiment in accordance with the Spanish and European regulations, under the animal experimentation project number 10514.

### Detection of CSFV RNA

CSFV RNA was extracted from sera, nasal, and rectal swabs, and tonsils. The tonsil tissue was homogenized in Eagles MEM (1 g tissue +9 ml medium) supplemented with penicillin 10,000 U/ml, streptomycin 10,000 µg/ml, and used for CSFV RNA detection. The MagAttract 96 *cador* Pathogen Kit (Qiagen) was used for RNA extraction following the manufacturer’s instructions, and the RNA was stored at −80°C until CSFV RNA analysis by quantitative reverse transcription-PCR (RT-qPCR) [32]. Cycle threshold (Ct) values of 40 and below were considered as positive. Ct values between 29 and 40 were considered as low, from 23 to 28 as moderate and from 10 to 22 as high viral RNA load as previously [33].

### CSFV rescue in tonsil samples from infected pigs

Tonsil samples were subjected to virus isolation in PK-15 cells. Briefly, the cells were seeded in 96-well plates for 24 hours before addition of 100 µL of 10- and 100-fold diluted samples. The virus was detected by PLA test after 72 hours of incubation [24]. Viral load was determined in the positive samples [19].

### Antibody detection by ELISA and virus neutralization assay

The serological analyses included the sera from the vPdR-H_30_K-36U-infected pigs of the present study and sera from pigs infected with vPdR-36U under the same conditions in a previous study [19]. ELISA was used to analyze the E2- and E^rns^-specific antibodies using the CSFV Ab test (IDEXX Laboratories, Liebefeld, Switzerland), and the pigtype CSFV E^rns^ Ab test (INDICAL), respectively, according to the manufacturer’s recommendations. For CSFV E2-specific antibodies, samples were considered positive when the blocking percentage was ≥40%. Likewise, CSFV E^rns^-specific Sample/Positive (S/P) values ≥0.5 were considered to be positive. Virus neutralization was determined using the neutralization peroxidase linked assay (NPLA) against the Alfort/187 strain with the sera samples that had scored positive by ELISA. The neutralization titers were expressed as the reciprocal dilution of serum that neutralized 100 TCID_50_ in the 50% of the culture replicates.

## Results

### The RNase activity of E^rns^ has no effect on the replication kinetics of the virus in cell culture

**Figure 1.**
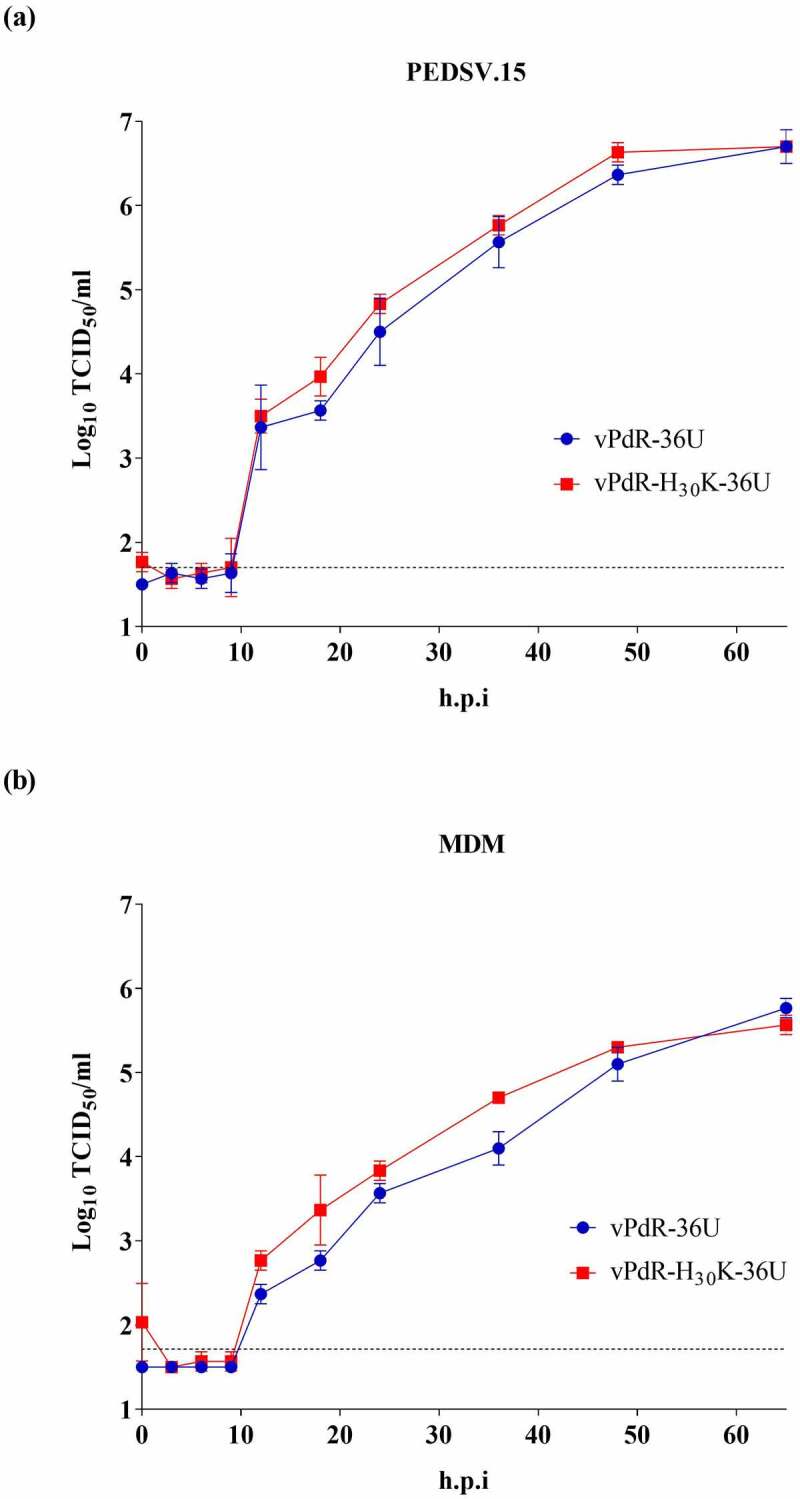
**Kinetics of virus replication in PEDSV.15 cells and porcine MDM**. PEDSV.15 cells and porcine MDM were infected in quadruplicate or in triplicate with vPdR-36U (in blue) or vPdR-H_30_K-36U (in red), respectively. The mean virus titer was determined in SK-6 cells at 3, 6, 9, 12, 18, 24, 36, 48 and 72 h.p.i and error bars show the standard deviation

The specific infectivity of the RNA transcripts and the titers of the rescued viruses were higher than 10^5^ focus forming units/µg RNA and 5 × 10^6^ TCID_50_/ml, respectively, in PEDSV.15 cells, which showed that the two cDNA clones were functional. The complete nucleotide sequence of the two viruses, vPdR-36U, and vPdR-H_30_K-36U, was determined after one additional passage in PEDSV.15 cells, confirming the expected E^rns^ sequence in the respective virus and excluding any other difference or accidental mutation. The growth characteristics of vPdR-H_30_K-36U and vPdR-36U were analyzed in PEDSV.15 cells (Figure 1(a)) and in porcine MDM (Figure 1(b)). No difference in the replication kinetics was found for the two viruses. This demonstrates that the RNase activity of E^rns^ does not influence the basic replication characteristics of the virus, neither in a porcine endothelial cell line nor in porcine MDM.

### The E^rns^ of vPdR-H_30_K-36U lacks RNase activity

**Figure 2.**
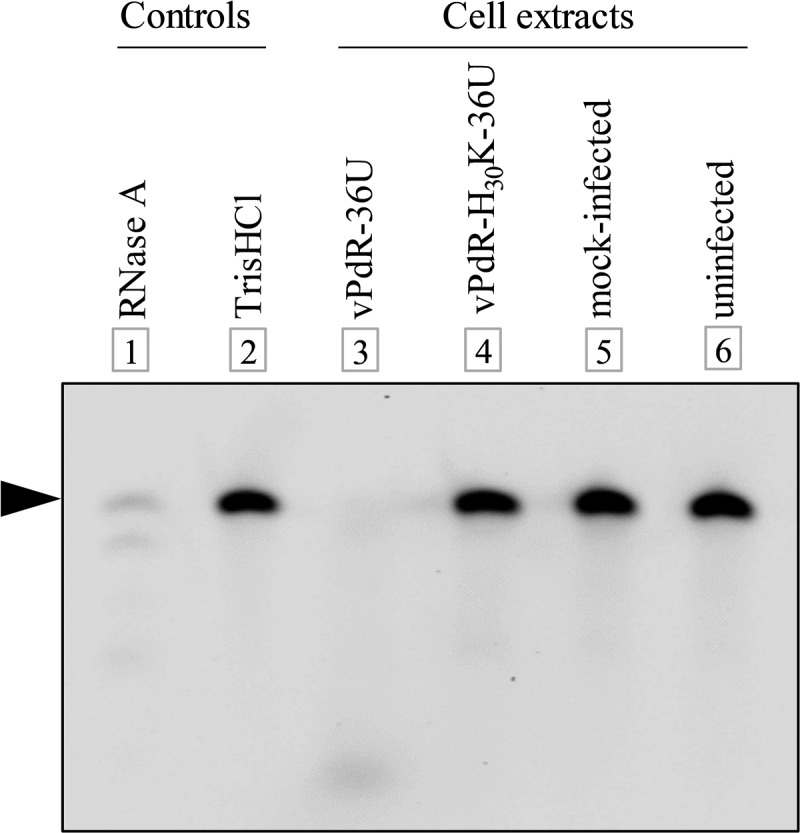
**RNase activity assay with vPdR-36U and vPdR-H_30_K-36U**. SK-6 cells were infected with vPdR-36U or vPdR-H_30_K-36U or mock or left uninfected. The cells were lysed after 44 hours and the extracts were incubated with the Dy-781-O1-RNA probe. The probe (black arrowhead) treated with the different cell extracts as indicated was separated by urea/polyacrylamide gel electrophoresis. Positive and negative controls were included in lanes 1 and 2, respectively

The histidine at amino acid position 30 of E^rns^ lies within the first catalytic domain of the RNase active site of E^rns^ [8]. We analyzed the E^rns^-specific RNase activity in cells infected with vPdR-36U and vPdR-H_30_K-36U and showed that the H_30_K mutation of E^rns^ abolished the CSFV-mediated degradation of an IRDye-labeled RNA probe completely (Figure 2). In contrast, the parent vPdR-36U virus digested the RNA probe at least as efficiently as the RNase A used as control (Figure 2). This confirms that substitution of the histidine codon 30 of E^rns^ with a lysine codon abolishes the RNase activity of the virus in infected cells as expected.

### The RNase-inactive vPdR-H_30_K-36U has lost the capacity to prevent IFN-α induction in porcine pDC

**Figure 3.**
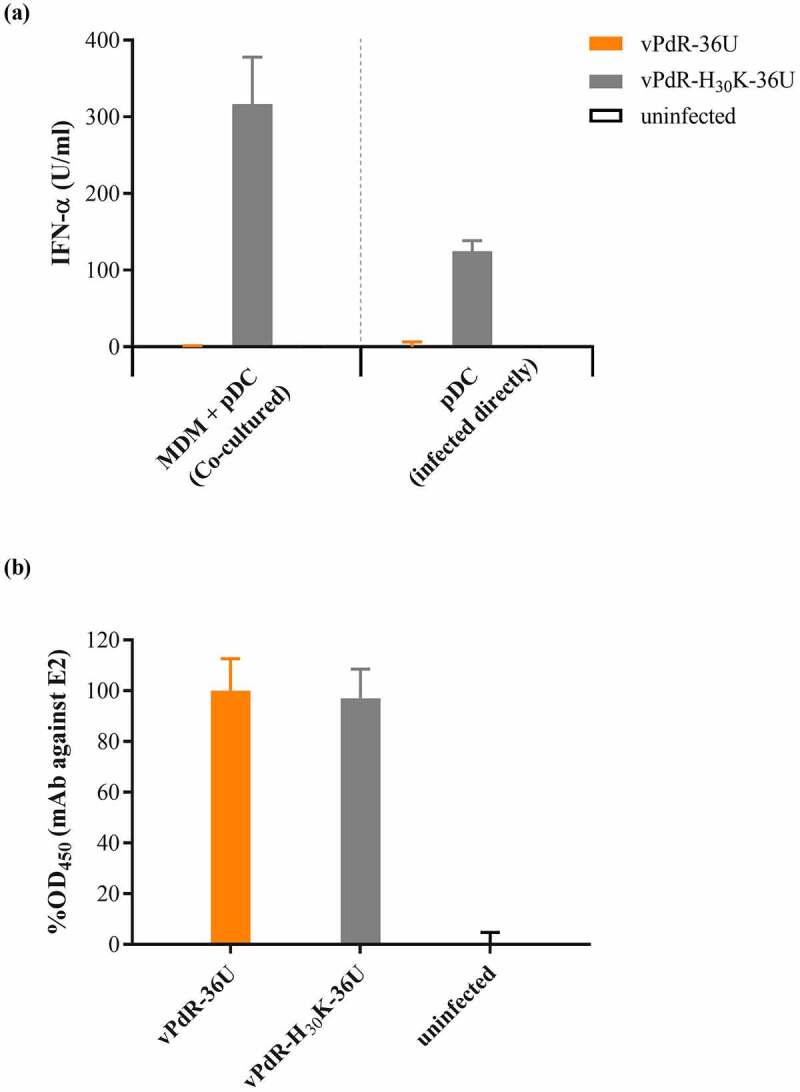
**IFN-α induction in pDC by co-culture with infected MDM or by direct infection with vPdR-36U or vPdR-H_30_K-36U**. (a) Mean IFN-α concentration in cell culture supernatants from enriched pDC co-cultured with uninfected MDM (white bars) or MDM infected with the vPdR-36U (orange bars) or vPdR-H_30_K-36U (gray bars, left panel) or from pDC infected directly with the indicated respective viruses (right panel). (b) The level of infection of the MDM by the respective viruses is shown as percentage of optical density (% OD) of the vPdR-36U signal obtained after immunoperoxidase staining of E2. Means and standard deviations (error bars) were calculated from six parallel cultures

It was shown previously that E^rns^ prevents pDC from producing IFN-α upon contact with CSFV-infected cells, which is dependent on the RNase activity E^rns^ [11]. Here we showed that MDM infected with vPdR-36U did not induce any detectable IFN-α in pDC brought in contact with them (Figure 3(a)). This contrasted with the vPdR-H_30_K-36U mutant that activated pDC efficiently for IFN-α production (Figure 3(a)). Importantly, the two viruses infected the MDM in contact with the pDC with the same efficiency (Figure 3(b)). Of note, CpG stimulation of parallel cultures of pDC resulted in 1420 IFN-α U/ml (±75), which confirms the functionality of the pDC for IFN-α production. This shows that the vPdR-H_30_K-36U as opposed to vPdR-36U activates the pDC for IFN-α production directly and in a cell-contact-dependent manner *in vitro*.

### Very mild clinical signs were observed in pigs infected with the E^rns^ RNase-inactive CSFV mutant

**Figure 4.**
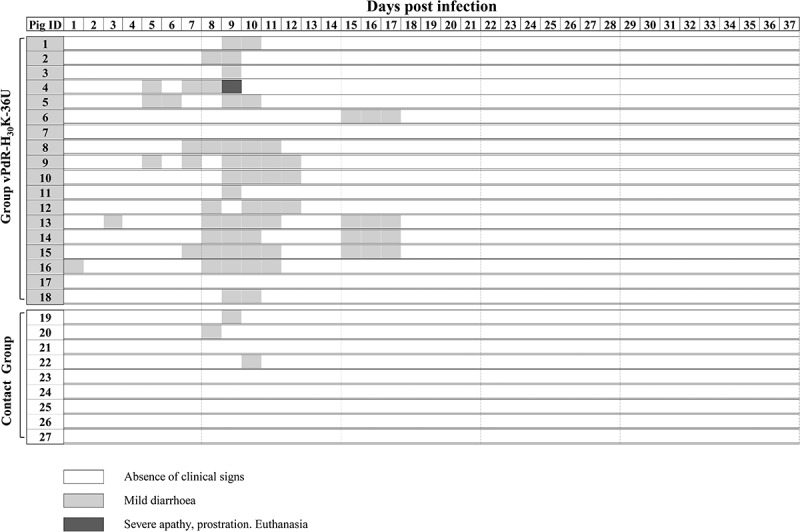
**Individual clinical signs evaluated after vPdR-H_30_K-36U infection**. Pigs 1 to 18 were inoculated with vPdR-H_30_K-36U and pigs 19 to 27 served as contact pigs introduced 24 hours post infection. The clinical signs were monitored daily during the complete study. Different shades of gray represent the severity of the clinical signs according to the legend

Mild diarrhea was the main clinical sign in the vPdR-H_30_K-36U-inoculated group (Figure 4). In general, diarrhea was detected during the second week of the study and did not last longer than 5 days in any case. From the third week on until the end of the trial, the animals were clinically healthy. However, one animal from the inoculated group had to be euthanized at 9 dpi after it had reached the endpoint criteria, despite showing only mild diarrhea the previous day. The contact pigs were clinically healthy during the entire trial, except for 3 out of 9 animals that showed sporadic mild diarrhea during 1 day in the second week of the study (Figure 4).

### The E^rns^ RNase-inactive CSFV mutant showed a very low replication capacity in pigs

**Figure 5.**
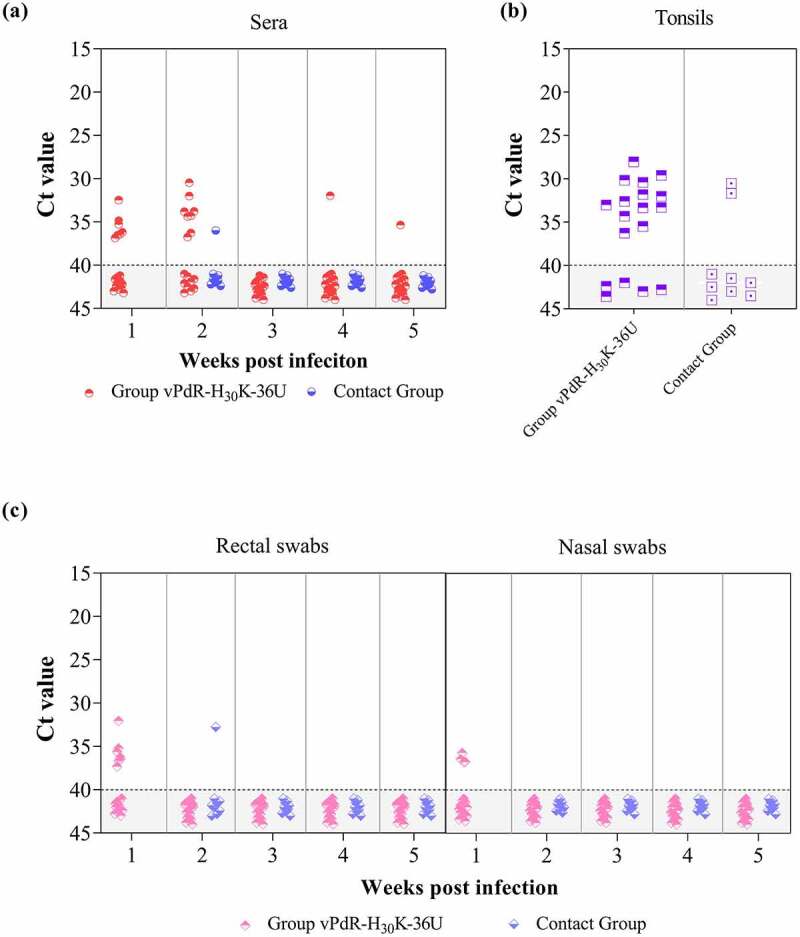
**Detection of CSFV RNA in sera, tonsils and swabs**. The CSFV RNA content was determined by RT-qPCR in the sera at weekly intervals (a) the tonsils obtained at necropsy (b) and the rectal and nasal swabs (c) collected on a weekly basis. Ct values higher than 40 (shaded area under the dotted line) were considered as negative

Low levels of CSFV RNA were detected in the sera from less than half of vPdR-H_30_K-36U inoculated piglets only, mostly during the first two weeks post infection, while the other piglets of the group remained negative (Figure 5(a)). Interestingly, from the third week onwards, nearly all infected animals were negative for viral RNA in the serum, with the exception of two samples, one at four (pig number 6) and another at five (pig number 3) weeks post infection (Ct value above 31.9). At necropsy, five out of 18 infected animals were negative for CSFV RNA in the tonsils. Low viral RNA load was found in 12 tonsil samples, the majority of them with Ct value above 30. Only one animal, *i.e*. pig number 4 that was euthanized at 9 dpi (Figure 4), showed moderate CSFV RNA levels in the tonsil with a Ct value of 28 (Figure 5(b)). However, no live virus could be isolated from any of the RT-qPCR positive tonsil samples.

### The E^rns^ RNase-inactive virus showed a very low capacity for excretion and transmission

Low virus shedding was observed in half of the infected pigs in the first week post infection only, as shown by Ct values above 30 in the nasal or rectal swabs. By contrast, the other half of infected animals remained negative during the whole trial (Figure 5(c)). Accordingly, virus transmission was observed only in one of the nine contact piglets (pig number 19) the second week of the study with a low RNA load in both, the serum and the rectal swab (Ct values higher than 32) (Figure 5(a,c)). In addition, two contact pigs were weakly positive for CSFV RNA in the tonsils but were again tested negative for virus isolation (Figure 5(b)).

### The CSFV E^rns^ RNase-negative mutant activates the IFN-α response in pigs

**Figure 6.**
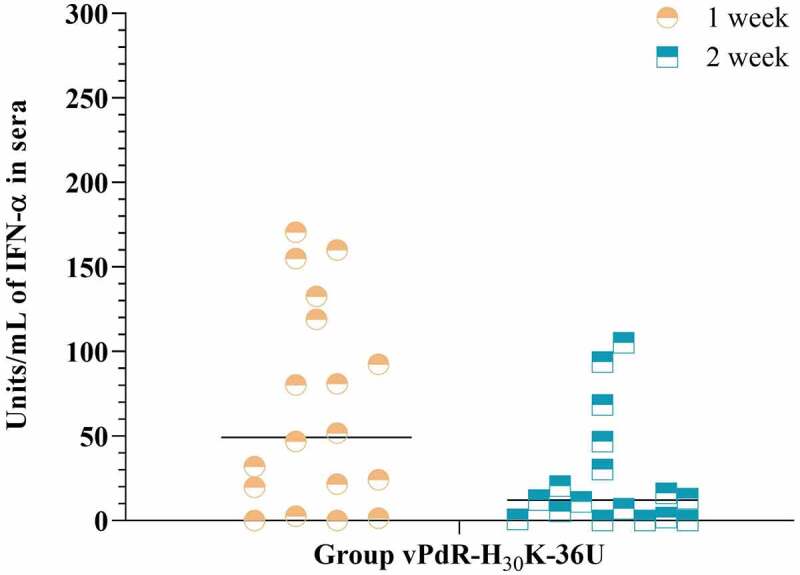
**IFN-α levels in sera during the first two weeks post infection**. Serum IFN-α levels were determined on the first (yellow symbols) and second (blue symbols) week after infection with vPdR-H_30_K-36U. The black lines represent mean values

The vPdR-H_30_K-36U-infected piglets had variable levels of IFN-α in the serum during the first two weeks post-infection while almost all the contact piglets remained negative. In the first week, 15 out of the 18 infected piglets were positive for IFN-α, five of which had serum IFN-α levels higher than 100 units/mL (Figure 6). The IFN-α levels were overall lower during the second week post infection. Six piglets had serum IFN-α below 25 units/mL or remained negative during the whole trial. Notably, in the contact group, only pig number 22 (which was also positive for CSFV RNA in the serum) had a high serum IFN-α level (153 units/mL) the second week of the study (data not shown).

### The absence of RNase activity of E^rns^ resulted in a more consistent E2-specific antibody response in pigs

**Figure 7.**
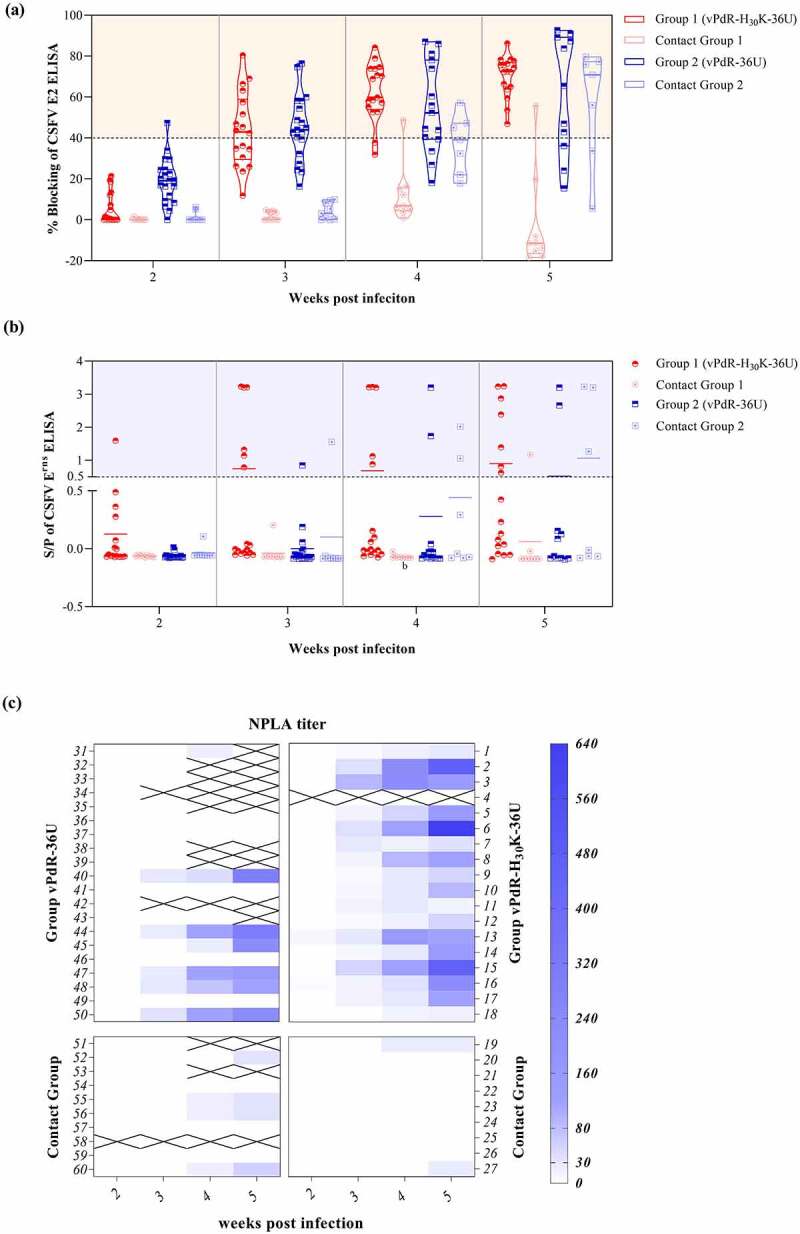
**Evaluation of the humoral immune response after vPdR-H_30_K-36U and vPdR-36U infection**. (a) The E2-specific antibody responses after infection with vPdR-H_30_K-36U and vPdR-36U are shown as percentage of blocking as measured by ELISA. Values between 30 and 40% of blocking were considered doubtful, and values equal or greater than 40% were considered positive. (b) The E^rns^-specific antibody responses after vPdR-H_30_K-36U and vPdR-36U infection are shown as S/P ratios as determined by ELISA. S/P ratios between 0.3 and 0.5 were considered doubtful, S/P ratios equal or greater than 0.5 were considered as positive. (c) The neutralizing antibody titers after vPdR-H_30_K-36U and vPdR-36U infection were determined by the NPLA at weekly intervals. Neutralizing antibody titers from low to high are represented on a scale from light to deep blue. Negative samples are shown in white. A cross shows that the animal was euthanized or dead

For the analysis of immune responses, we included serum samples from age-matched piglets infected with the parent RNase-positive vPdR-36U virus from our previous study using the same setup [19] and compared them with the sera from the vPdR-H_30_K-36U-infected piglets of the present study using ELISA and NPLA (Figure 7). In the second week post infection, only one vPdR-36U-infected piglet seroconverted against E2, while all other piglets from the two groups were still negative (Figure 7(a)). In the third and fourth weeks post infection, the E2 antibody responses of the two groups were similar, with only two E2-doubtful pigs for the vPdR-H_30_K-36U group and four negative or doubtful E2-specific responses for the vPdR-36U group on week 4. In the last week of the experiment, three of the surviving vPdR-36U-infected pigs were still E2-negative or doubtful by ELISA, while the rest were positive, especially for all piglets from the vPdR-H_30_K-36U group. The contact pigs of the vPdR-36U group seroconverted during the fourth week, with three positive animals out of seven. A week later, five of the seven remaining contact pigs were seropositive. In accordance with the lack of vPdR-H_30_K-36U transmission, the contact pigs from this group remained E2-negative, except for the single animal (number 22) that was infected by contact.

### The E^rns^ RNase-negative mutant induces enhanced E^rns^-specific antibody responses in pigs

In terms of E^rns^-specific antibody responses, only two of the pigs inoculated with vPdR-36U expressing functional E^rns^ RNase seroconverted against E^rns^, one on week 3, and the second on week 4. Consistent with the poor seroconversion against E^rns^ after vPdR-36U infection, only three out of 10 contact animals in this group were E^rns^ antibody-positive by the end of the study. Meanwhile, with the RNase-defective virus, seroconversion against E^rns^ started on the second week, and six animals had seroconverted against E^rns^ on the third week (Figure 7(b)). In accordance with the poor transmission of the vPdR-H_30_K-36U virus, only one contact pig became E^rns^ antibody-positive on the fifth week of the study (Figure 7(b)). Together, the RNase-negative PdR induced a more robust antibody response against E^rns^ while being less transmissible to contact animals, when compared with the parent PdR-36U virus carrying functional E^rns^.

### The RNase-defective vPdR-H_30_K-36U virus induced earlier and stronger neutralizing antibody responses than the parent virus

The neutralizing antibody responses induced by vPdR-36U *versus* vPdR-H_30_K-36U were compared by the NPLA test. During the first 2 weeks following the infection, none of the animals had neutralizing antibodies (Figure 7(c)). In the third week post infection, 16 out of the 17 vPdR-H_30_K-36U-infected pigs developed neutralizing antibodies, compared to only 5 out of the 18 survivors of the vPdR-36U group (Figure 7(c), top panels). At this time, in the latter group all neutralizing titers were below 1:30 (Figure 7(c), top left panel). In the fourth week, all vPdR-H_30_K-36U-infected piglets were positive, with neutralizing titers by up to 1:240. This contrasted with the vPdR-36U group in which only half of the survivors had neutralizing antibodies at this time, with maximum titers of 1:120. In the last week of the study, the neutralizing antibody titers further increased in all pigs of the vPdR-H_30_K-36U group, reaching 1:640. The neutralizing titers ranged from 1:120 to 1:320 in the survivors of the vPdR-36U group, with still half of the pigs lacking detectable neutralizing antibodies. In the contact animals of this latter group, titers ranged from 1:30 to 1:60. Low levels of neutralizing antibodies were found also during the fourth and fifth weeks of the trial in the two vPdR-H_30_K-36U animals that were infected by contact.

## Discussion

Nowadays, CSFV strains of low and moderate virulence continue to be prevalent in some endemic countries, making the CSF control particularly difficult [18,34]. However, data on the viral factors that determine attenuation and persistence remain scarce. The present study shows for the first time the effect of knocking out the RNase activity of E^rns^ in a low virulence CSFV strain, on viral replication, pathogenesis and transmission capacity in pigs. The results obtained here suggest a role of functional RNase activity of E^rns^ in dampening immune response activation and in the establishment of viral persistence in pigs. In accordance with previous studies showing that substitution or deletion of histidine residues within the catalytic domain of the E^rns^ RNase abolished RNase activity [6,8], the vPdR-H_30_K-36U mutant did not show any detectable E^rns^ RNase activity when comparted with its vPdR-36U parent. However, the RNase inactivation did not affect the viral replication in cell cultures, as vPdR-36U and vPdR-H_30_K-36U had similar replication characteristics in PEDSV.15 cells and in MDM. This is consistent with the fact that neither of the two viruses can induce any type I IFN in these latter cells as in any non-pDC, due to functional N^pro^ that mediates degradation of IFN regulatory factor 3 (IRF3) [11,35]. In direct contrast with this, when compared with vPdR-36U from a previous study [19], the E^rns^ RNase-negative vPdR-H_30_K-36U showed reduced or undetectable viral replication in pigs, as measured in sera, body secretions, and tonsils, in accordance with the very mild or even absence of clinical signs observed. Thus, the abrogation of the RNase activity in the low virulence PdR isolate had a strong attenuating effect *in vivo*, in agreement with previous studies with virulent CSFV or BVDV [8,9].

In addition, the low replication capacity *in vivo* of the E^rns^ RNase-negative virus resulted in a nearly complete loss of viral transmission of the virus to contact piglets. This was consistent with the absence of live virus in the nasal and rectal swabs, although traces of viral RNA were detectable by RT-qPCR with Ct values above 30. A previous study using the parental vPdR-36U showed that no live CSFV could be isolated from samples with Ct values above 32.2 [19]. In fact, only two contact piglets were infected with vPdR-H_30_K-36U, likely caused by infection via fomites rather than by pig-to-pig transmission, although the latter cannot be excluded. Notably, infection with vPdR-H_30_K-36U resulted in very low levels or absence of CSFV RNA in the tonsil, despite the general high capacity of CSFV to persist in the tonsils from infected or vaccinated animals for over 30 days [36]. This suggests that the lack of E^rns^ RNase activity may also reduce the capacity of CSFV to persist in the infected host. It should be noted here that the wild-type CSFV PdR isolate [16], with functional E^rns^ RNase activity is capable of generating persistently infected piglets, with absence of seroconversion as a hallmark [17]. In contrast, such persistence did not occur with the RNase-negative mutant under the same conditions in the present study. The vPdR-H_30_K-36U virus had reduced viral replication, with absence of infectious CSFV in the tonsils. These results are in line with the observation that the abrogation of the E^rns^ RNase activity in BVDV reduced the capacity to establish persistent infections in bovine fetuses [15].

Type I IFNs are key cytokines to establish an antiviral state and to potentiate the adaptive immune response [37]. In agreement with a previous study [11], the E^rns^ RNase-negative PdR mutant strongly activated the IFN-α production in pDC *in vitro* (Figure 3). This mutant also showed a clear activation of the IFN-α response in the infected pigs, however, with substantial differences between the animals. The serum IFN-α levels were lower when compared with those detected in a previous study, in pigs infected with the parent vPdR-36U virus expressing functional E^rns^ RNase [19]. Thus, the activation of IFN-α *in vivo* showed an inverse correlation with respect to the response observed in cell cultures [11], which could be explained by the different replication levels achieved in each system. The viral RNA-mediated trigger of IFN-α induction in the infected animals may be lower with the vPdR-H_30_K-36U due to the significantly lower replication than that reported previously for the parent vPdR-36U, which may not be compensated by the lack of the IFN antagonistic function of E^rns^ in pDC with the mutant [19,37]. These results support the role of CSFV E^rns^ RNase activity in the regulation of viral replication and in the modulation of innate immune activation in pigs.

Interestingly, the E^rns^ RNase-negative PdR mutant enhanced the adaptive immune response. High antibody levels against E2 and E^rns^, as well as an efficient neutralizing antibody response was detected in the vPdR-H_30_K-36U infected pigs. Notably, from four weeks post-infection onwards, the neutralizing antibody titers against non-homologous virus were above the threshold established for protection against CSFV infection [38] in nearly all the vPdR-H_30_K-36U-infected piglets. It was shown that CSFV-infected pigs can develop anti-E^rns^ antibodies as early as 10 dpi [39]. This has raised interest in E^rns^ as a potential diagnostic target for the differentiation of vaccinated from infected animals (DIVA) [39]. In the present study, we also evaluated the humoral response generated in pigs after infection with the vPdR-36U virus, using the sera collected previously [19]. The vPdR-36U is a cDNA-derived version of the field isolate Pinar del Rio (PR-11/10-3) from Cuba [16,23]. Remarkably, the vPdR-36U virus, failed to induce detectable E^rns^-specific antibody responses in piglets during five weeks of infection. In this regard, the abrogation of the E^rns^ RNase activity enhanced E^rns^-specific antibody response significantly, suggesting a better E^rns^ protein immunogenicity following the loss of the RNase activity. Therefore, the difficulties in the development of diagnostics and DIVA assays based on seroconversion against E^rns^ [40,41] may be attributable to a dampening effect on E^rns^-specific immunity related to the RNase activity of E^rns^. Further studies are required to clarify the role of the RNase activity of E^rns^ in the host immune modulation [11] by CSFV strains of different virulence.

These results pave the way towards a better understanding of CSFV attenuation related to the RNase function of E^rns^ through regulation of viral replication and immune responses in pigs. Our findings also provide new insights relevant for the development of DIVA vaccines against CSFV with appropriate accompanying diagnostic tests for efficient control and eradication of CSF and for the distinction from infections with other pestiviruses.

## Data Availability

The authors confirm that the data supporting the findings of this study are available within the article.
